# The WAG/Rij Rat Model of Depression Comorbid with Absence Epilepsy: Sex Differences and Neurochemical Mechanisms

**DOI:** 10.3390/ijms27052154

**Published:** 2026-02-25

**Authors:** Karine Yu. Sarkisova, Vladimir S. Kudrin

**Affiliations:** 1 Experimental Pathology of the Nervous System Group, Institute of Higher Nervous Activity and Neurophysiology, Russian Academy of Sciences, Moscow 117485, Russia; 2 Neurochemical Pharmacology Group, Federal State Budgetary Institution “Scientific Research Institute of Pharmacology named after V.V. Zakusov”, Moscow 125315, Russia

**Keywords:** depression, anxiety, sex difference, absence epilepsy, comorbidity, WAG/Rij rat, neurochemical mechanism, forced swimming test, sucrose preference, splash test

## Abstract

Depression is the most common psychiatric disorder and is frequently comorbid with epilepsy. Despite the high incidence of depression in epilepsy and the well-established sex differences in depression in clinical studies, sex differences in depression in epilepsy models remain unclear. This study aimed to investigate sex differences in depression comorbid with absence epilepsy and their neurochemical mechanisms in the WAG/Rij rat model. WAG/Rij rats, regardless of sex, exhibited symptoms of depression-like behaviour in the forced swimming test: increased immobility and decreased climbing, swimming, and diving. Both strain and sex differences were found in sucrose preference and splash tests, indicating anhedonia. However, anhedonia was more pronounced in WAG/Rij females compared to males. Unlike the males, WAG/Rij females showed signs of increased anxiety, suggesting an anxious depression phenotype. In WAG/Rij rats compared to Wistar controls, a reduced content of dopamine and its metabolites in brain structures was revealed, indicating a reduced dopaminergic tone of the brain. In WAG/Rij females compared to males, a more pronounced dopamine insufficiency and alterations in serotonin metabolism were found. The results indicate that sex differences in neurochemical alterations in brain structures may underlie sex differences in the manifestation of depressive pathology in the WAG/Rij rat preclinical model.

## 1. Introduction

Depression is the most common and heterogeneous psychiatric disorder, frequently comorbid with other psychiatric or neurological diseases, most often with epilepsy. The reported prevalence of depression in people with epilepsy ranges from 10.7 to 44%, reaching up to 54% in refractory epilepsy cases [1]. People diagnosed with depression have a significantly increased risk of subsequently developing epilepsy. Conversely, people with epilepsy also have a higher risk of developing depression [2]. These findings suggest that depression and epilepsy are not just comorbid, but they may share common underlying pathophysiological mechanisms [3,4,5]. The inability to experience pleasure from rewarding or enjoyable activities (anhedonia) is a core symptom of depression in humans. Current studies suggest that the pathophysiological mechanisms of depression and epilepsy comorbidity involve multiple factors, including chronic inflammation, neurotransmitter imbalance, hypothalamic–pituitary–adrenal (HPA) axis dysfunction, and impaired synaptic plasticity [6]. Among these factors, inflammation is considered a central mediator of the bidirectional relationship between epilepsy and depression [7]. Genetic, environmental, and epigenetic factors are suggested as etiological mechanisms of depression and epilepsy comorbidity.

The sex-related prevalence of major depressive disorder in the general population is approximately 1.7:1 in women compared to men. Similarly, the prevalence of anxiety, which is often comorbid with depression, is also higher in females (23.4%) than in males (14.3%) [8]. Epidemiological data indicate that female patients with epilepsy are more likely than males to develop depression. The incidence of depression in females with epilepsy is approximately 4.27 times higher than in males [9]. The prevalence of anxiety in people with epilepsy ranges from as low as 4.3% to as high as 52.1%. Anxiety is prevalent in 35.5% of females and in 23.2% of males. Comorbid anxiety has been found to exert a negative effect on seizure outcomes and quality of life in people with epilepsy [10]. Anxiety and depression in epilepsy represent a significant concern for epilepsy treatment because psychiatric comorbidities are associated with worse epilepsy outcomes [11]. Co-occurrence of epilepsy and psychiatric comorbidities is more likely associated with treatment resistance [12].

Despite the high incidence of psychiatric comorbidities in epilepsy and the well-established sex differences in anxiety and depression in the general population [13], there are few studies on sex differences in psychiatric comorbidities in epilepsy models [14]. The animal models used to study behaviour do not always offer consistent results with respect to sex differences observed in humans [15]. Increased anxiety- and depression-like behaviours have been observed in numerous experimental epilepsy models, including both genetic [16,17,18] and acquired epilepsy models [19,20,21,22]. However, none of these studies have examined sex differences in anxiety- or depression-like behaviours in epilepsy models. In one study, male and female genetically epilepsy-prone rats (GEPR-3s) and Sprague-Dawley controls were compared in a battery of tests sensitive to anxiety-like and depressive-like phenotypes. Most anxiety and depression-like measures showed significant differences between strains. GEPR-3s showed increased anxiety-like behaviour in the open-field test, elevated plus maze, light-dark transition test, and looming threat test. Moreover, GEPR-3s showed impaired prepulse inhibition of the acoustic startle reflex, decreased sucrose preference index, and impaired novel object recognition memory. However, only subtle sex differences (at the trend level) were found in head dips off the open arms and risk assessments in the elevated plus maze test. In summary, the findings suggest that the anxiety phenotype in this epilepsy model has a strong genetic component that may underlie both convulsive pathology and comorbidities seen in epilepsy [23].

It has been previously established that the Wistar Albino Glaxo/from Rijswijk (WAG/Rij) rat strain is a valid genetic animal model of childhood absence epilepsy, a non-convulsive form of epilepsy [24,25]. Subsequent studies have shown that WAG/Rij rats can also be considered a valid animal model of depression comorbid with absence epilepsy [25,26,27,28]. The model meets the necessary validity criteria to be an animal model of depression. The model shows similarities with depressive pathology in symptoms (face validity), response to conventional antidepressants (predictive validity), and neurochemical alterations in the brain (construct validity). So, epileptic WAG/Rij rats compared with non-epileptic Wistar rats exhibit behavioural depressive-like symptoms, such as decreased investigative activity in the open-field test, increased immobility in the forced swimming test, and reduced sucrose intake and preference (anhedonia). In addition, WAG/Rij rats adopt passive coping strategies in stressful situations, express submissiveness, and have a diminished ability to make choices and overcome obstacles, which are typical for depressed patients [27]. WAG/Rij rats are sensitive to chronic, but not acute, antidepressant treatments, suggesting that this rat strain fulfils a criterion of predictive validity to be an animal model of depression [17]. Reduced content of dopamine (DA) and its metabolites in the prefrontal cortex, nucleus accumbens, striatum, hypothalamus, and hippocampus, indicating a decreased DAergic tone, was found in WAG/Rij rats [29]. The DAergic insufficiency as a neurochemical mechanism of depression-like comorbidity in absence epilepsy is consistent with clinical data on the important role of DA and its receptors in the pathogenesis of depressive disorders in humans [30,31,32]. Decreased DAergic tone also plays an important role in the pathogenesis of absence seizures [33]. This means that decreased DAergic tone can be regarded as a common neurochemical mechanism of depressive-like behaviour and associated absence seizures. Recent studies have shown that early-life environment may contribute to the expression of a depression-like phenotype in adult offspring of WAG/Rij rats [34,35]. Early environmental manipulations also affect the manifestation of absence seizures in adult WAG/Rij rats [36]. These findings suggest that not only genetic but also environmental factors and epigenetic mechanisms may be involved in the phenotypic expression of absence seizures and comorbid depression in the WAG/Rij rat model [37]. In GAERS (Genetic Absence Epilepsy Rats from Strasbourg), another genetic model of absence epilepsy, elevated anxiety and depression-like behaviour were found. However, sex-related differences in the expression of these neuropsychiatric comorbid disorders in the GAERS absence epilepsy model have not been investigated. It should be noted that neuropsychiatric comorbidities in the WAG/Rij rat model have previously been studied using only males [17,26,37]. Even though some behavioural traits of female WAG/Rij rats have been described previously [38], the problem of sex-related differences in depression comorbid with absence epilepsy, as well as in the content of monoamines and their metabolites in brain structures as their possible neurochemical mechanisms, remains unresolved.

This study aimed to find out the sex differences in the manifestation of depression-like comorbidity in absence epilepsy and their underlying neurochemical mechanisms in the WAG/Rij rat preclinical model.

## 2. Results

### 2.1. Sex Differences in the Level of Anxiety in WAG/Rij and Wistar Rats

#### 2.1.1. Light–Dark Choice Test

**Figure 1:**
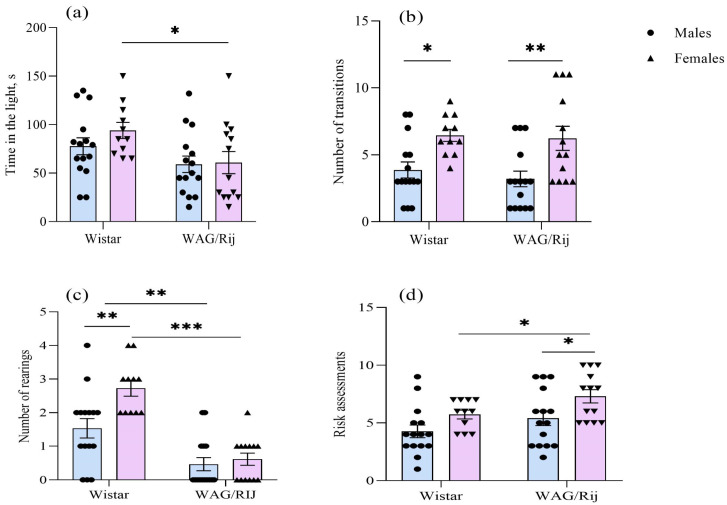
Behavioural measures of males and females of Wistar and WAG/Rij rats in the light–dark choice test. (**a**) Time in the light, s; (**b**) number of transitions between compartments; (**c**) number of rearings in the light compartment; (**d**) number of risk assessments. Values are the mean ± S.E.M. The individual data points are displayed in bar graphs to show within-group variability. Wistar males (*n* = 15), Wistar females (*n* = 11), WAG/Rij males (*n* = 15), WAG/Rij females (*n* = 13). ***—*p* < 0.001, **—*p* < 0.01, *—*p* < 0.05. Horizontal lines indicate the groups being compared.

In the light–dark choice test, the two-way ANOVA showed a significant effect of strain on the time spent in the light [F(1,50) = 7.6, *p* < 0.01]; the effects of the factor “sex” and the interaction of the factors “strain” and “sex” were insignificant. In WAG/Rij and Wistar rats, no significant sex differences were found in the time spent in the light. In WAG/Rij females, the time in the light was shorter than that in Wistar females (Figure 1a). The effect of sex was significant for the number of transitions [F(1,50) = 17.6, *p* < 0.001]. In females of both strains, the number of transitions between the light and dark compartments was greater than in males (Figure 1b).

The number of rearing behaviours was affected by the factors “strain” [F(1,50) = 45.7, *p* < 0.001] and “sex” [F(1,50) = 8.2, *p* < 0.01] and the interaction of these factors [F(1,50) = 4.9, *p* < 0.05]. The number of rearing behaviours in Wistar females was greater than in males. WAG/Rij rats of both sexes exhibited fewer rearings than Wistar rats (Figure 1c). The two-way ANOVA revealed a significant effect of the factors “strain” [F(1,50) = 5.7, *p* < 0.05] and “sex” [F(1,50 = 8.8, *p* < 0.01] on the risk assessments. It was greater in WAG/Rij females compared to Wistar females and WAG/Rij males (Figure 1d).

#### 2.1.2. Open-Field Test

**Figure 2:**
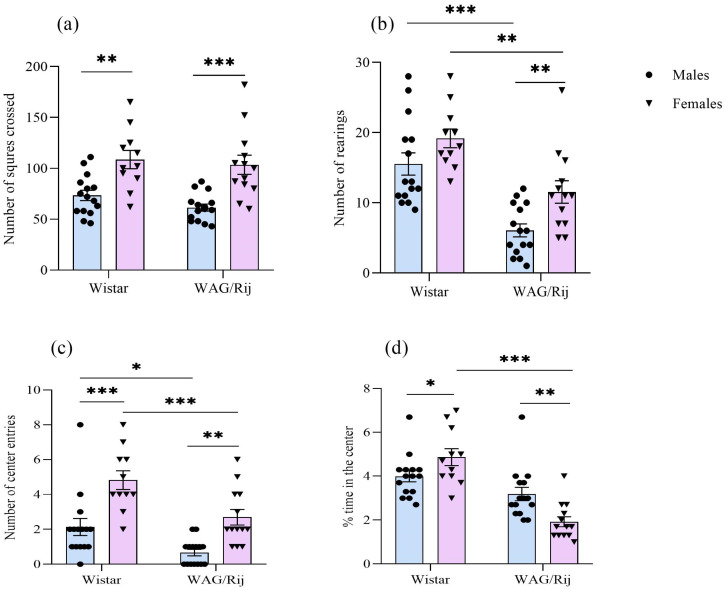
Behavioural measures of males and females of Wistar and WAG/Rij rats in the open-field test. (**a**) Number of squares crossed; (**b**) number of rearings; (**c**) number of centre entries; (**d**) % time in the centre. Values are the mean ± S.E.M. The individual data points are displayed in bar graphs to show within-group variability. Wistar males (*n* = 15), Wistar females (*n* = 11), WAG/Rij males (*n* = 15), WAG/Rij females (*n* = 13). ***—*p* < 0.001, **—*p* < 0.01, *—*p* < 0.05. Horizontal lines indicate the groups being compared.

In the open-field test, the two-way ANOVA showed a significant effect of the factor “sex” on the number of squares crossed [F(1,50) = 32.3, *p* < 0.001]. The number of squares crossed was greater in females of both strains compared with males (Figure 2a). The factors “strain” [F(1,50) = 37.3, *p* < 0.001] and “sex” [F(1,50) = 10.6, *p* < 0.01] were significant for the number of rearings. This behavioural measure was greater in WAG/Rij females compared to WAG/Rij males. In WAG/Rij rats of both sexes, the number of rearing behaviours was less compared with Wistar rats (Figure 2b). The number of centre entries was affected by the factor “strain” [F(1,50) = 17.9, *p* < 0.001] and “sex” [F(1,50) = 30.8, *p* < 0.001]. The interaction of these factors was not significant. The number of centre entries was greater in females of both strains. However, in WAG/Rij males and females, this behavioural measure was lower compared with the corresponding values in Wistar males and females (Figure 2c).

Percentage time in the centre of the open field was affected by the factor “strain” [F(1,50) = 40.8, *p* < 0.001] and the interaction of the “strain” and “sex” factors [F(1,50) = 13.3, *p* < 0.001]. In Wistar rats, % time in the centre of the open field was greater in females than in males. In contrast, in WAG/Rij females, this measure was lower than in WAG/Rij males (Figure 2d).

The factors “strain” [F(1,50) = 57.5, *p* < 0.001] and “sex” [F(1,50) = 11.5, *p* < 0.01] were significant for the number of groomings. The number of grooming reactions was greater in females than in males of both strains, but less in WAG/Rij rats of both sexes compared to Wistar rats.

### 2.2. Sex Differences in Depression-like Behaviour in WAG/Rij and Wistar Rats

#### 2.2.1. Forced Swimming Test

**Figure 3:**
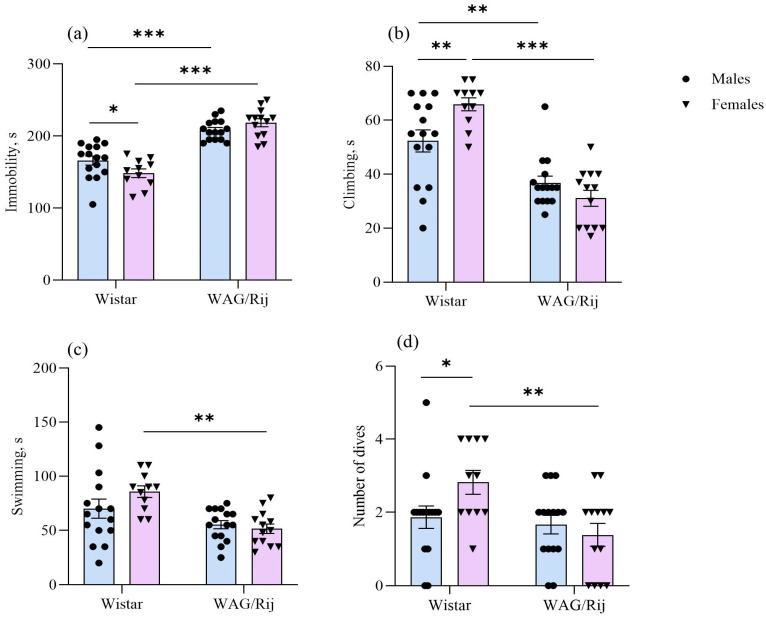
Behavioural measures of males and females of Wistar and WAG/Rij rats in the forced swimming test. (**a**) Immobility time, s; (**b**) the duration of climbing, s; (**c**) the duration of swimming, s; (**d**) number of dives. Values are the mean ± S.E.M. The individual data points are displayed in bar graphs to show within-group variability. Wistar males (*n* = 15), Wistar females (*n* = 11), WAG/Rij males (*n* = 15), WAG/Rij females (*n* = 13). ***—*p* < 0.001, **—*p* < 0.01, *—*p* < 0.05. Horizontal lines indicate the groups being compared.

The two-way ANOVA showed a significant effect of strain [F(1,50) =106.8, *p* < 0.001] and the interaction of the strain and sex factors [F(1,50) = 6.6, *p* < 0.05] on the immobility time in the forced swimming test. WAG/Rij rats of both sexes exhibited longer immobility time than the corresponding groups of Wistar rats. In female Wistar rats, immobility time was shorter compared with that of males of the same strain (Figure 3a).

The duration of climbing was affected by the factor “strain” [F(1,50) = 62.2, *p* < 0.001] and the interaction of the factors “strain” and “sex” [F(1,50) = 9.1, *p* < 0.01].

The duration of climbing was longer in Wistar females than in males. This behavioural measure was shorter in WAG/Rij rats of both sexes compared to Wistar rats (Figure 3b).

The factor “strain” was significant for the duration of swimming [F(1,50) =15.4, *p* < 0.001]. In female Wistar rats, the duration of swimming was insignificantly (*p* = 0.8) longer than in Wistar males. In WAG/Rij females, this behavioural measure was significantly shorter (*p* < 0.01) than in Wistar females (Figure 3c). The number of dives (an indicator of active coping strategy) was affected by the factor “strain” [F(1,50) = 7.4, *p* < 0.01] and the interaction of the factors “strain” and “sex” [F(1,50) = 4.2, *p* < 0.05]. The factor “sex” was insignificant. The number of dives in Wistar females was greater than in Wistar males, but in WAG/Rij females it was less than in Wistar females (Figure 3d). The strain and sex factors did not affect the number of boli.

#### 2.2.2. Sucrose Consumption and Preference Test

The analysis of covariance (ANCOVA) with body weight as a covariate showed that the contribution of this covariate to the total variance of any of the dependent variables in the sucrose consumption test was not significant. Therefore, this covariate could not explain the differences between strains and sex regarding the depression-like phenotype. This implied that the effects of two factors (strain and sex) could be analyzed with a classical ANOVA.

**Figure 4:**
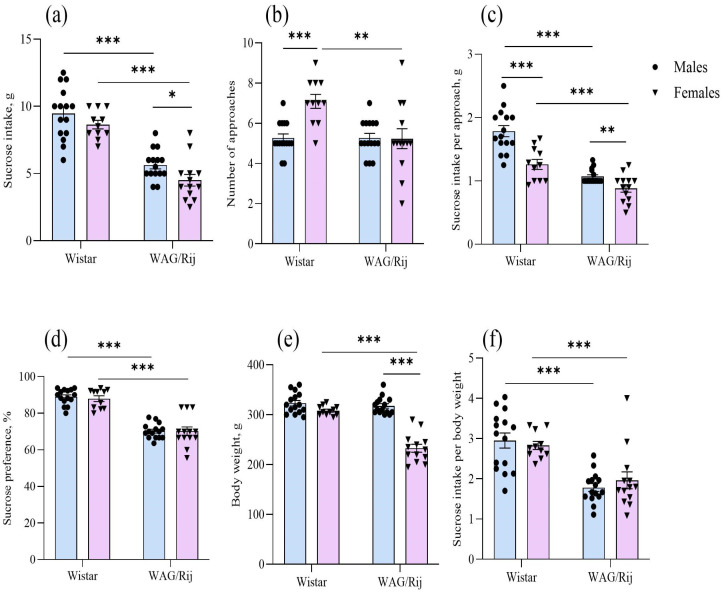
Behavioural measures of males and females of Wistar and WAG/Rij rats in the sucrose consumption and preference test. (**a**) Sucrose intake, g; (**b**) number of approaches to the sucrose bottle; (**c**) sucrose intake per approach to the sucrose bottle; (**d**) sucrose preference, %; (**e**) body weight, g; (**f**) sucrose intake per body weight. Values are the mean ± S.E.M. The individual data points are displayed in bar graphs to show within-group variability. Wistar males (*n* = 15), Wistar females (*n* = 11), WAG/Rij males (*n* = 15), WAG/Rij females (*n* = 13). ***—*p* < 0.001, **—*p* < 0.01, *—*p* < 0.05. Horizontal lines indicate the groups being compared.

The two-way ANOVA showed a significant effect of strain [F(1,50) = 95.9, *p* < 0.001] and sex [F(1,50) = 5.8, *p* < 0.05] on the amount of sucrose consumed. In males and females of WAG/Rij rats, sucrose intake was less than in Wistar rats. In addition, female WAG/Rij rats consumed less sucrose than male WAG/Rij rats [F(1,26) = 5.0, *p* < 0.05] (Figure 4a). The number of approaches to the bottle with sucrose was affected by the factors “strain” [F(1,50) = 8.1, *p* < 0.01] and “sex” [F(1,50) = 7.5, *p* < 0.01], and the interaction of these factors [F(1,50) = 9.1, *p* < 0.01]. In Wistar females, the number of approaches to the sucrose bottle (an indirect indicator of explorative activity during the test) was greater than in Wistar males. In WAG/Rij females, the number of approaches to the sucrose bottle was less than in Wistar females (Figure 4b). The effects of strain [F(1,50) = 65.2, *p* < 0.001], sex [F(1,50) = 27.5, *p* < 0.001] and strain and sex interaction [F(1,50) = 6.2, *p* < 0.05] were significant for sucrose intake per approach (a hedonic index of sucrose palatability or ‘‘liking’’). In WAG/Rij rats of both sexes, the amount of sucrose consumed per approach was less than in Wistar rats. In Wistar females, this measure was significantly less (*p* < 0.001) than in Wistar males, as well as in WAG/Rij females (*p* < 0.01) compared to males (Figure 4c).

Sucrose preference (%) was affected by the factor “strain” [F(1,50) = 135.7, *p* < 0.001]. The factor “sex” and the interaction of the factors “strain” and “sex” were insignificant. Sucrose preference in WAG/Rij males and females was less than in Wistar rats of both sexes, indicating inter-strain differences in anhedonia (Figure 4d).

Body weight was affected by the factors “strain” [F(1,50) = 51.5, *p* < 0.001] and “sex” [F(1,50) = 78.4, *p* < 0.001] and the interaction of these factors [F(1,50) = 38.8, *p* < 0.001]. The body weight of female WAG/Rij rats (232.7 ± 7.9 g) was significantly (*p* < 0.001) lower than that of male WAG/Rij rats (317.7 ± 4.4 g) and female Wistar rats (308.2 ± 2.9 g). In Wistar rats, the body weight of females (308.2 ± 2.9 g) was also lower than that of males (323.0 ± 5.4 g), but this difference did not reach statistical significance (Figure 4e). The factor “strain” was significant for sucrose intake per body weight [F(1,50) = 39.3, *p* < 0.001]. In WAG/Rij males and females, sucrose intake per body weight was less than in Wistar rats of both sexes (Figure 4f).

**Figure 5:**
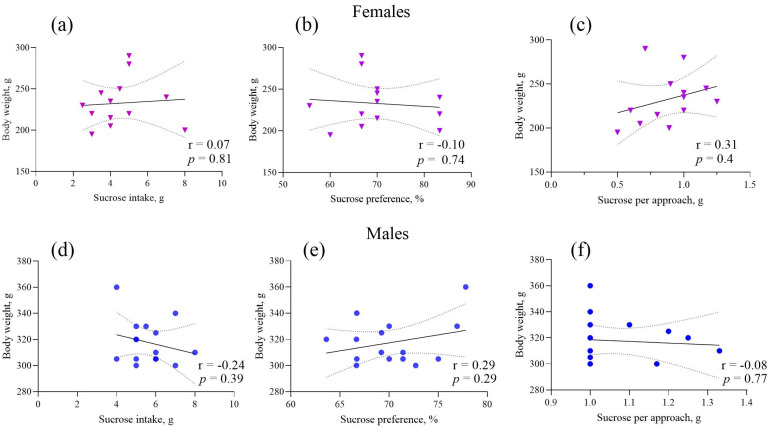
Correlations between body weight and sucrose consumption/preference test measures in WAG/Rij females (**a**–**c**) and WAG/Rij males (**d**–**f**). All correlations were insignificant.

In addition to the analysis of covariance (ANCOVA), Pearson correlation coefficients were computed. We found no statistically significant correlations between body weight and sucrose consumption/preference test measures (Figure 5).

#### 2.2.3. Splash Test

**Figure 6:**
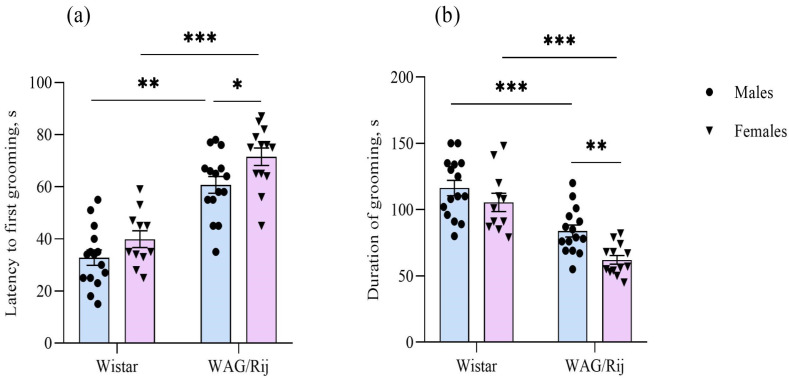
Behavioural measures of males and females of Wistar and WAG/Rij rats in the splash test. (**a**) Latency to first grooming, s; (**b**) duration of grooming, s. ***—*p* < 0.001, **—*p* < 0.01, *—*p* < 0.05. Horizontal lines indicate the groups being compared.

In the splash test, two-way ANOVA revealed a significant influence of strain [F(1,50) = 85.7, *p* < 0.001] and sex [F(1,50) = 7.8, *p* < 0.01] factors on the latency to first episode of grooming behaviour (Figure 6a) and also strain [F(1,50) = 52.9, *p* < 0.001] and sex [F(1,50) = 9.7, *p* < 0.01] factors on the duration of grooming (Figure 6b). The post hoc Newman–Keuls test showed that the latency to first grooming in WAG/Rij rats of both sexes is longer than in their Wistar counterparts. Moreover, in WAG/Rij females, latency to first grooming was greater and the duration of grooming was shorter than in WAG/Rij males, pointing to a reduced motivation for self-care as one of the forms of anhedonia and depressive-like apathetic behaviour.

### 2.3. Sex Differences in Brain Neurochemistry in WAG/Rij and Wistar Rats

Biochemical data have shown that the most pronounced inter-strain and sex differences are observed in the content of noradrenaline (NA), dopamine (DA) and its metabolites (DOPAC and HVA) in the nucleus accumbens, striatum and hypothalamus. Less pronounced strain and sex differences were also found in the content of NA and DA in the prefrontal cortex. The indices of DA turnover, DOPAC/DA and HVA/DA ratios, did not differ statistically significantly between the strains or between the sexes in all brain structures. Significant strain differences were also found in the 5-HT content, its metabolite 5-HIAA, and the 5-HIAA-to-5-HT ratio in the hippocampus, nucleus accumbens, and prefrontal cortex (Table 1. The results of post-hoc analysis are presented in Supplementary Table S1).

In the prefrontal cortex, the two-way ANOVA revealed a significant effect of the factor “strain” [F(1,46) = 5.3, *p* < 0.05] and “sex” [F(1,46) = 8.2, *p* < 0.01] on the content of NA; the interaction of these factors was insignificant. In WAG/Rij females, the content of NA was lower than in WAG/Rij males. The content of NA in WAG/Rij males was greater compared to their Wistar counterparts. ANOVA also showed a significant effect of the factor “strain” on the level of DA [F(1,46) = 9.9, *p* < 0.01]. In WAG/Rij males, the DA level was lower compared to Wistar males. The content of 5-HIAA, the primary product of the enzymatic degradation of serotonin by monoamine oxidase A (MAO-A), was affected by the factor “strain” [F(1,46) = 8.4, *p* < 0.01].

The effect of the factor “sex” was insignificant. In WAG/Rij females, the level of 5-HIAA was higher than in Wistar females. The 5-HIAA-to-5-HT ratio, which is an indication of the serotonin turnover rate and serves as an estimated index for assessing the rate of release of 5-HT into the synapse, was also affected by the factor “strain” [F(1,46) = 17.9, *p* < 0.001]. The factor “sex” was insignificant. In WAG/Rij males and females, the 5-HIAA-to-5-HT ratio was greater than in Wistar females.

In the nucleus accumbens, the factor “strain” was significant for the content of NA [F(1,46) = 8.9, *p* < 0.01]. In WAG/Rij males, this measure was greater than in Wistar males. The factor “sex” and the interaction of the factors “strain” and “sex” were insignificant. The effects of “strain” [F(1,46) = 15.7, *p* < 0.001] and “sex” [F(1,46) = 12.6, *p* < 0.001] were significant for the level of DA and its metabolite DOPAC [F(1,46) = 7.1, *p* < 0.01] and [F(1,46) = 9.8, *p* < 0.01], respectively. In females of both strains, the concentration of DA was less than that in males. The content of DA was smaller in WAG/Rij males and females compared to their Wistar counterparts. The level of DOPAC was lower in Wistar females and WAG/Rij males compared to Wistar males. The content of HVA was affected by the factors “strain” [F(1,46) = 8.0, *p* < 0.01] and “sex” [F(1,46) = 7.4, *p* < 0.01], but the interaction of these factors was insignificant. In Wistar females, the level of HVA was lower than in Wistar males, and in WAG/Rij males, it was lower than in Wistar males. The factor “sex” was significant [F(1,46) = 16.8, *p* < 0.001] for the content of 3-methoxytyramine (3-MT), but the factor “strain” was insignificant. In females of both strains, the content of 3-MT was less compared with that of males. The factor “strain” influenced the DOPAC-to-DA ratio [F(1,46) = 8.4, *p* < 0.01] and the HVA-to-DA ratio [F(1,46) = 8.2, *p* < 0.01]. However, the post hoc test did not reveal statistically significant strain or sex differences. The level of HIAA was affected by the factor “strain” only [F(1,46) = 10.7, *p* < 0.01]. In WAG/Rij males, the level of HIAA was higher than in Wistar males. The factor “strain” was significant for the 5-HIAA-to-5-HT ratio [F(1,46) = 8.5, *p* < 0.01]. In WAG/Rij females, this measure of serotonin turnover was greater than in Wistar females.

In the striatum, the content of NA was affected by the interaction of the factors “strain” and “sex” [F(1,46) = 6.7, *p* < 0.05]. The effects of the factors “strain” and “sex” were insignificant. In WAG/Rij females, the content of NA was smaller than in WAG/Rij males. The DA level was affected by the factor “strain” [F(1,46) = 23.3, *p* < 0.001] and “sex” [F(1,46) = 8.2, *p* < 0.01]. The content of DA was smaller in Wistar females compared to males, and in WAG/Rij males and females compared to their Wistar counterparts. The effect of the factor “strain” was significant for the DOPAC level [F(1,46) = 33.0, *p* < 0.001]; the effect of the factor “sex” was insignificant [F(1,46) = 3.7, *p* = 0.06]. The DOPAC level was smaller in Wistar females compared to Wistar males and in WAG/Rij males and females compared to Wistar rats. The content of HVA was also affected by the factor “strain” [F(1,46) = 28.2, *p* < 0.001], and the effect of the factor “sex” was insignificant [F(1,46) = 3.7, *p* = 0.59]. In males and females of WAG/Rij rats, the HVA level was lower compared to Wistar males and females. The content of 3-MT was influenced by the factors “strain” [F(1,46) = 9.0, *p* < 0.01] and “sex” [F(1,46) = 15.7, *p* < 0.001], but the interaction of these factors was insignificant. In Wistar females, the level of 3-MT was lower than in Wistar males, but in WAG/Rij females, the level of 3-MT was higher than in Wistar females and lower than in WAG/Rij males. No significant strain or sex differences were found in the content of 5-HT and 5-HIAA and the 5-HT/5-HIAA ratio.

In the hypothalamus, the factors “strain” [F(1,46) = 17.8, *p* < 0.001] and “sex” [F(1,46) = 27.2, *p* < 0.001] were significant for the content of NA; the interaction of these factors was insignificant. The level of NA was lower in females of both strains compared with males, but it was higher in WAG/Rij males and females compared with Wistar rats. The content of DA was affected by the factor “strain” [F(1,46) = 10.9, *p* < 0.01] and the interaction of the factors “strain” and “sex” [F(1,46) = 6.8, *p* < 0.05]. The factor “sex” was insignificant [F(1,46) = 2.9, *p* = 0.09]. In Wistar females and WAG/Rij males, the level of DA was lower than in Wistar males. The DOPAC content was affected by the factor “strain” [F(1,46) = 21.3, *p* < 0.001]. The effects of the factor “sex”, as well as the interaction of the factors “strain” and “sex”, were insignificant. In Wistar females and WAG/Rij males, the level of DOPAC was lower than in Wistar males, but in WAG/Rij females, it was lower than in Wistar females [F(1,24) = 5.8, *p* < 0.05]. The 3-MT content was affected by the factor “strain” [F(1,46) = 4.5, *p* < 0.05] and the interaction of the factors “strain” and “sex” [F(1,46) = 5.0, *p* < 0.05]. The effect of the factor “sex” was insignificant. In WAG/Rij females, the level of 3-MT was higher than in Wistar females. No significant strain or sex differences were found in the content of HVA, the DOPAC/DA ratio, the HVA/DA ratio, 5-HT, 5-HIAA and the 5-HIAA/5-HT ratio.

In the hippocampus, the two-way ANOVA showed no statistically significant strain or sex differences in the content of NA, DA and its metabolites (DOPAC, HVA, 3-MT). Strain and sex differences in this brain structure were found only in the 5-HT content, its metabolite 5-HIAA, and the 5-HIAA-to-5-HT ratio. The factor “strain” was significant for the content of 5-HT [F(1,46) = 17.1, *p* < 0.001]. The factor “sex” and the interaction of the factors “strain” and “sex” were insignificant. In WAG/Rij males and females, the 5-HT content was smaller than in Wistar males and females. The level of 5-HIAA was affected by the factor “sex” [F(1,46) = 4.2, *p* < 0.05] and the interaction of the factors “strain” and “sex” [F(1,46) = 5.0, *p* < 0.05]. In Wistar females, the content of HIAA was greater than in males, and in WAG/Rij males, this measure was greater than in Wistar males. The 5-HIAA/5-HT ratio was affected by the factor “strain” [F(1,46) = 41.3, *p* < 0.001]; the factor “sex” was insignificant [F(1,46) = 3.0, *p* = 0.09]. In WAG/Rij males and females, the 5-HIAA/5-HT ratio was greater than in Wistar males and females.

Neurochemical data presented in Table 1 indicate that male and female WAG/Rij rats, compared with Wistar rats, show the same (reciprocal) changes in the activity of the DAergic and serotonergic brain systems. This is evidenced by decreased levels of DA and its metabolites in the nucleus accumbens, striatum, and hypothalamus, as well as decreased levels of 5-HT in the hippocampus and elevated levels of the 5-HIAA-to-5-HT ratio (the indicator of 5-HT metabolism) in the prefrontal cortex and hippocampus. A similar decrease in the activity of the DAergic system (reduced DOPAC level) and an increase in the activity of the NAergic system (elevated level of NA) were also found in male and female WAG/Rij rats in the hypothalamus (Table 2).

**Figure 7:**
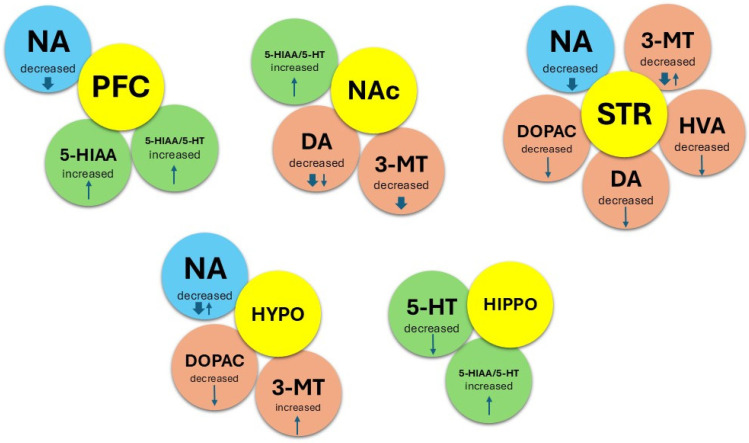
Schematic representation of sex differences in the content of monoamines and their metabolites in brain structures of WAG/Rij females. **↓**—decreased, **↑**—increased. Bold arrow—WAG/Rij females compared to WAG/Rij males; thin arrow—WAG/Rij females compared to Wistar females.

In addition, female WAG/Rij rats also have changes in brain neurochemistry compared to male WAG/Rij rats (Figure 7). In female WAG/Rij rats, the activity of the DAergic brain system is reduced not only in comparison with Wistar females (indicated by thin arrows), but also in comparison with Wistar males (indicated by bold arrows). Thus, the levels of DA and 3-MT in the nucleus accumbens, 3-MT and NA in the striatum, and NA in the prefrontal cortex and hypothalamus were lower in WAG/Rij females compared to males, indicating not only a more pronounced deficiency of the DAergic system, but also a decreased activity of the NAergic system.

## 3. Discussion

We have previously shown that male WAG/Rij rats exhibit depression-like behaviour, such as increased immobility in the forced swimming test and decreased sucrose intake and preference (anhedonia) compared with male Wistar rats [17,26,27]. The results of the present study confirm and extend these data. It has been demonstrated that WAG/Rij females, like WAG/Rij males, also exhibit behavioural symptoms of depression. However, the symptoms of depressive-like behaviour, according to some behavioural measures, were more pronounced in WAG/Rij females than in WAG/Rij males. So, in the sucrose consumption test, the total amount of sucrose consumed and the amount of sucrose consumed per approach to the sucrose bottle (a hedonic indicator of sucrose palatability or ‘liking’) [39] in WAG/Rij females was less than in WAG/Rij males and Wistar females, pointing to a more pronounced anhedonia.

While the present study showed a decreased consumption of sucrose solution in WAG/Rij females, as indexed by the total amount of solution ingested and the amount of sucrose consumed per approach to the drinking bottle, a potential confound arises from the fact that WAG/Rij females were of lower body weight than their age-matched male counterparts. When accounting for body weight (sucrose intake/body weight), sex differences between WAG/Rij females and males became insignificant. However, despite the lower body weight in WAG/Rij females compared to WAG/Rij males and Wistar females, the analysis of covariance (ANCOVA) for all dependent variables (sucrose consumption, sucrose consumed per approach to the drinking bottle, and sucrose preference) with body weight as a covariate showed that the contribution of this covariate to the total variance was not significant. Therefore, this covariate could not explain the differences between strains and sexes concerning the depression-like behavioural profile. In other words, it means that both male and female WAG/Rij rats exhibit anhedonia, a key symptom of depression-like behaviour, compared to Wistar controls. Moreover, there were no significant correlations between body weight and sucrose consumed per approach to the drinking bottle, both in WAG/Rij females (r = 0.31, *p* = 0.40) and males (r = −0.08, *p* = 0.77). No correlations were found between body weight and the amount of sucrose consumed (r = 0.07, *p* = 0.81 for WAG/Rij females; r = −0.24, *p* = 0.39 for males) and sucrose preference (r = −0.10, *p* = 0.74 for WAG/Rij females; r = 0.29, *p* = 0.29 for males). Because the amount of sucrose consumed per approach to the drinking bottle is an indication of the palatability of the hedonic response to the solution being consumed [39], this measure can be considered an analogue of consummatory anhedonia [40], as seen in human depression [41,42]. In other words, in WAG/Rij rats, along with strain-associated differences in anhedonia, there are also sex-related differences. Female WAG/Rij rats show a more pronounced consummatory anhedonia compared to male WAG/Rij rats. The splash test data confirmed this conclusion. Firstly, WAG/Rij rats exhibited a longer latency to first grooming and a shorter duration of grooming compared with their Wistar counterparts, which indicates strain differences. Secondly, these anhedonia measures in WAG/Rij females differed from those in WAG/Rij males, suggesting sex differences: a more pronounced anhedonia or apathetic-like behaviour [43] in females. Strain and sex-related differences in the splash test, another endophenotype of depression, were also observed in different strains of mice [44].

Sex differences in the forced swimming test have been widely reported in many rat strains. However, the findings are not consistent across different studies. Both active and passive stress-coping strategies have been reported in females compared to males [45,46]. In the present study, Wistar females were more active than males in the forced swimming test, which is consistent with the literature data [47]. Wistar females showed a lower level of immobility, more climbing and swimming and a greater number of dives, indicating an active coping strategy. In WAG/Rij rats compared with Wistar rats, only strain but not sex differences were found. Both male and female WAG/Rij rats, compared to Wistar rats, showed passive stress-coping strategies or depression-like behaviour in the forced swimming test. They exhibited a greater level of immobility and less climbing. However, WAG/Rij females were more passive compared to Wistar females. WAG/Rij females demonstrated a greater immobility, less climbing, less swimming and a reduced number of dives. This is an indicator of a more pronounced inability to cope with stress, which is characteristic of a depression-like phenotype.

In the open-field test, the exploratory activity (the number of rearing behaviours and centre entries) in WAG/Rij females, as in WAG/Rij males, was lower than in their Wistar counterparts. These data are in line with those we obtained earlier using WAG/Rij males only [17,26,48]. At the same time, the level of locomotor and exploratory activity (the number of squares crossed and centre entries, respectively) in females of both strains was higher compared to males. These data are consistent with the data from literature, indicating that females consistently show higher activity levels than males in the open-field test [47,49]. In other words, the sex differences typical of other strains of rats (females are more active than males) remain in WAG/Rij rats. However, the differences in open-field activity between females and males of the WAG/Rij strain were greater compared to the differences between females and males of the Wistar strain. For example, the mean number of squares crossed, rearings, and centre entries in female WAG/Rij rats exceeded the corresponding values in males by ~1.7, 1.9, and 3.8 times, respectively, while in female Wistar rats, these values exceeded those in males by ~1.2, 1.2, and 2.2 times, respectively. This may indicate that female WAG/Rij rats are more prone to hyperactivity in a new environment than female Wistar rats.

The number of centre entries, as well as the relative frequency of crossing of the centre of the open field in female WAG/Rij rats, was greater than in males of the same strain. However, the relative time (%) spent in the centre of the open field in WAG/Rij females was ~2 times less than in males of the same strain and ~5 times less than in Wistar females, indicating increased anxiety in female WAG/Rij rats. The increased anxiety in female WAG/Rij rats has also been found in the light-dark choice test. Female WAG/Rij rats showed differences from males of the same strain and from females of Wistar rats in all light-dark test measures that characterize anxiety in this test (latency to enter the dark compartment, time spent in the light compartment, the number of rearing behaviours in the light compartment and the number of risk assessments). In male WAG/Rij rats, the time spent in the light and the number of risk assessments did not differ from the same behavioural measures in Wistar rats. This means that there are no distinct differences in anxiety levels between WAG/Rij males and Wistar males. These findings are consistent with those we published earlier [27,48].

Literature data indicate that females of many strains of rats and mice exhibit increased activity compared to males. It is believed that the greater motor and exploratory activity in females compared to males is a result of increased functional activity of the DAergic system in the striatum and the nucleus accumbens, which is caused by the action of sex hormones, primarily estrogen [50,51,52]. Interestingly, unlike in females, estrogen does not affect the level of DA in the striatum in males. Thus, ovariectomy reduced the concentration of DA in the striatum, as well as motor activity in female rats. Castration did not affect either DA levels in the striatum or motor activity in males [50]. It is assumed that there are two mechanisms by which estrogen causes an increase in the activity of the nigrostriatal and mesolimbic DAergic systems. One of them acts by inhibiting GABAergic neurons that form recurrent collaterals at DAergic terminals. The second one acts through direct action on DAergic terminals, causing increased DA release by acting on presynaptic D2-autoreceptors [50].

Females differ from males both at the level of DA synthesis and release and at the level of its reuptake and reception (density of D1 and D2 receptors), which probably leads to their different sensitivity to the effects of psychostimulants and antipsychotics [50,51,52,53]. Preclinical studies suggest that the higher D1–D2 heteromer expression in females may significantly increase predisposition to depressive-like and anxiety-like behaviours in female animals [54]. Similar differences in the activity of the DAergic brain system were also found in women, which may explain their differences from men in their greater predisposition to alcohol and drug addiction, as well as to many psychiatric disorders, particularly depression, as evidenced by clinical data [13,55,56].

Previous behavioural and neurochemical studies have demonstrated functional deficits in the brain DAergic system responsible for depression-like behaviour in WAG/Rij rats [27,29,57]. A reduced DAergic tone of the mesolimbic brain system was shown to be associated with both depression-like behaviour [29] and comorbid absence seizures [33] in WAG/Rij rats. In the present study, significant strain and sex differences were found in the content of DA and its metabolites in brain structures responsible for the manifestation of depression-like behavioural symptoms (the prefrontal cortex, nucleus accumbens, striatum, and hypothalamus). In WAG/Rij males compared to Wistar males, a decreased level of DA was observed in the prefrontal cortex, nucleus accumbens, striatum, and hypothalamus. This indicates a reduced DAergic tone in the brain, which we previously described in males of WAG/Rij rats [29,57]. Compared to Wistar females, WAG/Rij females, like males, also showed reduced activity in the DAergic brain system. This means that similar alterations in the functional activity of the DAergic brain system in male and female WAG/Rij rats compared to Wistar rats (Table 2) may underlie a similar depressive-like behavioural profile described in this study. However, in WAG/Rij females, compared to males, lower levels of DA and 3-MT, a marker of released DA [58], were found in the nucleus accumbens (Figure 7), a key brain structure known to be involved in reward, motivation, and depression-like behaviours [59,60,61,62]. These sex-related biochemical changes (lower DA and 3-MT levels in the nucleus accumbens), indicating a more pronounced insufficiency of the mesolimbic DAergic system, may explain the more pronounced anhedonia (in sucrose consumption/preference and splash tests) in female WAG/Rij rats compared to WAG/Rij males. This assumption is confirmed by the presence of a positive correlation between sucrose preference and DA content in the nucleus accumbens, which we established earlier in WAG/Rij rats [63]. In addition, microdialysis experiments have shown a direct relationship between the content of DA in the nucleus accumbens and the amount of sucrose consumed as a function of sucrose solution concentration. Higher sucrose intake at a higher concentration was associated with higher levels of DA in the nucleus accumbens and vice versa [64]. Decreased levels of striatal DA and hippocampal 5-HT were also found in female WAG/Rij rats compared to males. The decreased activity of striatal DAergic and hippocampal 5-HTergic systems may contribute to the manifestation of depressive-like symptoms in female WAG/Rij rats by affecting reward processing, leading to reduced motivation and anhedonia. Reduced levels of NA in the prefrontal cortex and hippocampus can also contribute to the manifestation of anhedonia (apathetic behaviour) in females of WAG/Rij rats. Interestingly, depressive-like behaviour caused by intranigral injections of 6-OHDA was accompanied by striatal DA and hippocampal 5-HT reductions. Moreover, DA and 5-HT levels were correlated with behavioural measures in the sucrose preference and forced swimming tests, suggesting the participation of striatal DA and hippocampal 5-HT in anhedonia and stress-coping strategies in the forced swimming test [65]. In our previous studies, a positive correlation was found between the duration of the first episode of active swimming (climbing, struggling) in the forced swimming test and the content of DA in the striatum [63]. This correlation probably reflects the relationship between the concentration of DA in the striatum and the intensity of the motor response to forced swimming stress (strong movements of the front paws, jumping). In other words, the level of DA in the striatum may underlie a stress-coping strategy (active or passive) in the forced swimming test. A positive correlation between hippocampal 5-HT and swimming behaviour in the forced swimming test was also found [65]. Therefore, the passive coping strategy (reduced active behaviours) in the forced swimming test in WAG/Rij rats may be explained by a reduced DA content in the striatum and 5-HT content in the hippocampus.

Despite some conflicting data, multiple lines of evidence suggest that dysfunction of the mesocorticolimbic DAergic system, especially hypofunction, can lead to hyperactivity caused by hypersensitivity to environmental stimuli [66,67]. It can be assumed that in female WAG/Rij rats, a low level of tonic DA release causes an increased level of phasic DA release, leading to locomotor hyperactivity as a manifestation of hypersensitivity of the DAergic brain system. This assumption is supported by previously obtained data on the hypersensitive locomotor response to novelty and amphetamine, a DA-releasing agent, in WAG/Rij rats, indicating higher DA reactivity of the mesolimbic system [68].

The results of this study indicate that there are sex differences in the manifestation of depression-like pathology in WAG/Rij rats as a genetic animal model of depression comorbid with absence epilepsy. Female WAG/Rij rats, like male WAG/Rij rats, show distinct behavioural symptoms of depression. However, female WAG/Rij rats, in contrast to male WAG/Rij rats, exhibit more pronounced signs of increased anxiety, anhedonia, and predisposition to hyperactivity in a novel, stressful environment. These behavioural symptoms are characteristic of a subtype of depressive disorders, such as anxious depression. Overall, our findings confirm and expand the previously obtained data concerning the behavioural characteristics of female WAG/Rij rats [38]. Epidemiological evidence suggests that anxious depression is a common and clinically relevant subtype of depression in humans [69]. Anxious depression is known to have a different neurobiological profile compared to non-anxious depression and is more prevalent in women [70]. Anxious depression in humans is defined as a psychiatric disorder in which symptoms of anxiety and depression are both present, but neither is clearly predominant. WAG/Rij females exhibit behavioural symptoms of both depression and anxiety without a clear predominance of symptoms of one of the components of a single phenotype. This may indicate that female WAG/Rij rats meet the face validity criterion to be an animal model of anxious depression comorbid with absence epilepsy. Both depression and anxiety disorders are suggested to be caused by impairments in multiple neurotransmitter systems and a complexity of circuits. In women, the gonadal hormones play a vital role in the regulation and functioning of neurochemical systems in the brain. It is believed that hormonal impact on neurotransmission may partially explain the greater predisposition to depression in general and to anxious depression, in particular, in women. There is evidence that an imbalance of the neurochemical systems, especially DAergic and 5-HTergic, may underlie anxious depression in humans [70]. The imbalance of the neurochemical systems was also shown in WAG/Rij rats. Changes not only in the DAergic system but also in the 5-HTergic system were found. In the hippocampus, decreased 5-HT levels and increased 5-HIAA/5-HT ratios were observed in both male and female WAG/Rij rats. However, increased 5-HIAA levels in the prefrontal cortex and increased 5-HIAA/5-HT ratios in the prefrontal cortex and nucleus accumbens were observed only in WAG/Rij females compared to Wistar females. 5-HTergic activity has been implicated in anxiety and depression. Therefore, it can be assumed that the dysfunction of not only the DAergic but also 5-HTergic system can underlie the anxious depression phenotype [71] in females of WAG/Rij rats. It has been suggested that sex differences in the functioning of the 5-HTergic brain system may affect predisposition to depression and the effectiveness of antidepressant treatment. Some reports indicate that selective serotonin re-uptake inhibitors are more effective in women, while tricyclic antidepressants are more effective in men. However, sex differences in antidepressant response remain unclear. It can be assumed that the different effectiveness of antidepressant therapy in women and men may be partly due to sex differences in brain neurochemistry. The question of whether there are sex differences in the effects of antidepressant therapy in male and female WAG/Rij rats awaits further research.

Multiple data suggest sex differences in human epilepsies. Specifically, childhood and juvenile absence epilepsy are more common in females compared with males [14]. Sex differences in pre-clinical absence epilepsy models have been studied in WAG/Rij [72,73] and GAERS [74]. In WAG/Rij rats, as shown in earlier studies, the number and duration of spike-wave discharges (SWDs) did not differ between the sexes [72]. In GAERS, similar to WAG/Rij rats, no sex-related differences in absence seizures were found [74]. In contrast to previous reports, longitudinal EEG registration in WAG/Rij rats has shown a consistent age-related increase in the absence seizure susceptibility (increases in the number, mean, and total duration of SWDs) in males compared with females [75]. Although absence seizures did not differ in GAERS, sex differences were found in social behaviour: females, in contrast to males, demonstrated deficits in sociability [76].

It should be noted that most pre-clinical animal models of depression were developed using male rats and mice, as well as the WAG/Rij rat model of depression comorbid with absence epilepsy [27]. The data obtained in this study expand the practical significance of the previously developed WAG/Rij rat model. The results of the present study suggest the potential utility of WAG/Rij females, similar to males, as a pre-clinical animal model of behavioural depressive-like symptoms associated with absence epilepsy.

One limitation of this study is the lack of verification of the estrous cycle phase in female rats and its impact on behavioural test measures. Previously, it was believed that the estrous cycle causes significant behavioural variability in female rodents, which may have led to the less frequent use of females in behavioural studies [77]. However, subsequent studies have shown that the behaviour of females, regardless of their estrous cycle, is not more variable than that of males in anxiety tests [78,79,80]. Furthermore, a number of studies failed to detect an effect of the estrous cycle on behavioural test measures in females [81]. No convincing evidence was found to support the notion that female behaviours in the open field, forced swimming, social interaction, and resident-intruder tests are influenced by the estrous cycle. Only minimal impact of the estrous cycle on the tests used was found [82]. The authors suggested that “different effects of the estrous cycle (in the forced swimming test) between studies are probably related to different methodological approaches and factors influencing the behavioural responses” [46]. Given that estrous cycles in female rats change rapidly over a short (4-day) period, and that behavioural testing in our study using a battery of tests took significantly more time (6–7 days), the outcome of a particular test could depend on the phase of the cycle in which the test was conducted. Moreover, the assessment of the female estrous cycle phase may have caused more handling of females than males, which is known to affect the behavioural outcome, including immobility in the forced swimming test and sucrose preference test. The examination of the estrous cycle not only increased depression-like behaviours, but also impaired short-term and long-term memory, indicating that this procedure is stressful [83]. The handling-induced stress can cause changes in subsequent behavioural test outcomes, altering or even masking the true effects of the estrous cycle itself. Not only behavioural testing but also the procedure itself for the examination of the estrous cycle phase in females could have an even greater impact on the brain neurochemistry in WAG/Rij rats. Therefore, considering the specifics of our experiments, the assessment of the estrous cycle phase was not performed in this study. However, the question of whether the stages of the estrous cycle affect the outcome of the behavioural tests used in these experiments is the subject of a separate special study, which may be the topic of our further research.

In summary, the WAG/Rij rat model can be used to study sex differences in depression comorbid with absence epilepsy, and its underlying mechanisms, and to develop new treatment strategies based on sex differences in the manifestation of depressive-like pathology. Female WAG/Rij rats, as well as male WAG/Rij rats, can be particularly useful models for assessing the sex-related therapeutic efficacy of various medications, including antidepressants, for the treatment of depression-like impairments associated with absence epilepsy. Understanding the biological basis of sex differences in neuropsychiatric comorbidities of absence epilepsy is likely to be a useful approach to the mechanisms of this comorbidity. Given the importance of sex differences in various neuropsychiatric and neurological diseases, testing WAG/Rij females is critical to identify sex-related effects on disease manifestation and treatment responsiveness. The different manifestations of comorbid depression in WAG/Rij males (non-anxious depression) and WAG/Rij females (anxious depression) make it possible to investigate the neurobiological and molecular mechanisms underlying the differences between anxious and non-anxious depression using the same genetic absence epilepsy model. Moreover, our findings indicating sex differences in the DAergic and 5-HTergic brain systems suggest sex-specific targets or treatment strategies, which may have potential translational significance.

## 4. Materials and Methods

### 4.1. Animals

Six-month-old males (*n* = 15) and females (*n* = 13) of inbred WAG/Rij rats and age-matched males (*n* = 15) and females (*n* = 11) of Wistar rats were used for behavioural studies; 12 males and 13 females of the WAG/Rij strain and 12 males and 13 females of the Wistar strain were used for biochemical analyses. WAG/Rij rats were born and raised at the Institute of Higher Nervous Activity and Neurophysiology of the Russian Academy of Sciences (IHNA). They represented approximately the 70th generation from parents originally obtained from Radboud University Nijmegen (Nijmegen, The Netherlands). Wistar rats aged 1–1.5 months were purchased from the breeding company “Stolbovaya”, and then raised at the IHNA in the same vivarium conditions as WAG/Rij rats. All rats were kept in a 12/12 h light–dark cycle (lights on at 8.00 a.m.). The animals were housed in standard plastic cages, with 3 to 4 animals per cage. Food and tap water were available ad libitum. Experiments were conducted in accordance with the European Union Directive 2010/63/EU on the protection of animals used for scientific purposes. The institutional policies and guidelines confirm animal care and use. Experimental protocols were approved by the Ethical Committee of the IHNA (protocol number 2 of 21 March 2024). Every effort was made to minimize the number of animals used in experiments and their suffering from experimental procedures, according to the 3Rs concept.

### 4.2. Behavioural Testing: Anxiety Level and Depression-like Symptoms

The males and females of Wistar and WAG/Rij strains were compared for differences in behavioural tests relevant to anxiety and depression. The level of anxiety was determined in the light–dark choice and open-field tests. The sucrose consumption/preference test, splash test, and forced swimming test were used to assess sex differences in depression-like behavioural alterations in the WAG/Rij rats compared with the Wistar controls. All experimental sessions were performed during the light phase (beginning at 10.00 a.m.). The male and female rats were tested on different days to exclude the effect of scent marks during behavioural testing.

#### 4.2.1. Light–Dark Choice Test

The apparatus consisted of two compartments with openings between them. The large compartment (36 cm × 18 cm) was light (100 lx), and the small one (18 cm × 17 cm) was dark (<5 lx). Each rat was placed in the light compartment facing away from the opening. The following behavioural reactions were measured for 5 min: latency of entering the dark compartment, the time spent in each compartment, the number of transitions between compartments (a measure of locomotor activity), the number of rearing instances and the number of risk assessments (aborted attempts to enter the light compartment). The shorter the time spent in the light compartment, the lower the number of transitions between compartments and rearing, and the greater the number of risk assessments (an ethologically relevant measure of anxiety), the higher the level of anxiety in the test, and vice versa [17,84].

#### 4.2.2. Open-Field Test

The open-field test was used to assess locomotor activity and anxiety-like behaviour. The apparatus was a circular arena, 100 cm in diameter, with a 30 cm wall and a floor divided into 32 squares by five vertical and five horizontal lines. Four squares were taken as the ‘‘centre’’ of the field. The test room was dimly lit (40 W) to decrease the aversiveness of the test. An animal was placed in the centre of the field (80 lx), and the following variables were recorded for 5 min: time to leave the centre, latency to the first visit to the centre, the number of squares crossed, rearing, and centre entries, % time in the centre, grooming reactions, and faecal boli (defecation). The open field was cleaned with 70% alcohol to remove odour marks each time after testing a rat. Low ambulatory activity, a reduced number of entries into the centre of an open field, and a short amount of time spent in it are commonly interpreted as a high level of anxiety, and vice versa [48,85,86].

#### 4.2.3. Sucrose Consumption/Preference Test

Each rat was placed in a test cage identical to the home cage. The fluid intake (consumption of 20% sucrose solution, g), the number of approaches to the drinking bottle (an indirect indicator of exploratory activity during the test), and the amount of sucrose consumed per approach (a hedonic index of sucrose palatability or ‘‘liking’’) [39] were recorded for 15 min [17,48]. Sucrose intake was measured by reweighing a pre-weighed bottle at the end of the test. Before testing, animals were not food- or water-deprived. In our previous experiments, it was found that a stable level of sucrose consumption could be achieved in rats of both strains by the 4th to 5th sessions of exposure [26,48]. Therefore, in this study, rats were pre-exposed to the sucrose consumption test for 4 days (adaptation to the procedure). The values of fluid intake on the 5th day were used for statistical evaluation of differences between groups of rats. Sucrose intake is a measure of anhedonia, which is widely used to assess an animal’s sensitivity to reward (the ability to experience pleasure) [87,88]. Decreased sucrose consumption and preference, that is, anhedonia, is considered to be a well-validated index of a depressive-like state in animals [88,89], homologous to that of depressed patients [90]. In the sucrose preference test, the animals were exposed to both the test solution (20% sucrose) and drinking water for a period of 1 h [26,48]. Preference (%) for sucrose (PS) over water, the most reliable hedonic index, was calculated as PS = [(sucrose intake, g/total fluid intake, g) × 100]. The sucrose preference test was carried out on the 6th day, one day after the last sucrose consumption test [26].

#### 4.2.4. Splash Test

The next day after the sucrose preference test, a splash test was performed in the same cage. The splash test was used to evaluate the grooming behaviour caused by spraying a 10% sucrose solution on the dorsal surface of the animal’s fur [44,91]. After sucrose solution application, the latency to first grooming and the duration of grooming (nose/head washing and body grooming) were recorded for five minutes as an index of motivation for self-care. It is believed that decreased motivation for self-care is one of the forms of anhedonia and depressive-like apathetic behaviour [43].

#### 4.2.5. Forced Swimming Test

The forced swimming test is a standard test to measure depression-like behaviour in experimental animals. This test is widely used to assess the antidepressant potential of various pharmacological drugs. The forced swimming test for the assessment of stress coping (“behavioural despair” or depression-like behaviour) was modified from the test initially described by Porsolt [92]. Only one test session without a pre-test was used [17,26,35]. The apparatus was a cylinder (height 47 cm, inside diameter 38 cm) containing 38 cm of tap water maintained at 24 °C. A video camera recorded the behaviour of rats in the forced swimming test. Rats were individually forced to swim. The following behavioural measures were recorded for 5 min: the duration of passive swimming (immobility), the first episode of active swimming (climbing), the duration of swimming, and the number of dives and boli. Immobility was defined as no movements by the rat (floating vertically in the water, front limbs immovable and clasped to the breast, and the nose kept above the water surface). Climbing (“struggling”, jumping) was defined as upward-directed strong movements of the front limbs that resemble scratching the wall of the container. All other less vigorous movements on the water surface throughout the water tank, with the rat in a horizontal position, were defined as swimming [17,26]. The increased immobility and decreased active behaviours (climbing, swimming, diving) in this test are considered to be measures of a depression-like phenotype [93,94]. The forced swimming test (Porsolt’s test of “behavioural despair”) is stressful for animals, so it was the last test performed to prevent any effect on the level of anxiety.

### 4.3. Brain Monoamines and Their Metabolite Content Measurements

The content of monoamines and their metabolites was assessed in males and females of WAG/Rij and Wistar rats in 5 brain structures: the prefrontal cortex, nucleus accumbens, striatum, hippocampus and hypothalamus. Animals intended for biochemical studies were not subjected to behavioural testing to prevent any possible effects of testing on the brain monoamines and their metabolite levels. The rats were decapitated with a guillotine for laboratory animals. Then, the brain structures (prefrontal cortex, hippocampus, striatum, nucleus accumbens, and hypothalamus) were removed on ice, frozen in liquid nitrogen, and weighed. The samples were stored in liquid nitrogen and later assayed for levels of monoamines and their metabolites using high-pressure liquid chromatography (HPLC) with electrochemical detection. The details of the method used have been described earlier [27,57,94]. Briefly, the brain tissue was homogenized (Potter homogenizer, glass-Teflon) in 1.0 mL of 0.1 M HClO_4_ containing 3,4-dihydroxy benzylamine (0.5 nmol/mL) as an internal standard and centrifuged at 10,000× *g* for 10 min at 4 °C. The supernatant (20 µL) was removed and analyzed by HPLC to determine the concentration of noradrenaline (NA), dopamine (DA), 3,4-dihydroxyphenylacetic acid (DOPAC), homovanillic acid (HVA), 5-hydroxytryptamine or serotonin (5-HT), and 5-hydroxyindolacetic acid (5-HIAA). Chromatograph LC-304 T (BAS, West Lafayette, IN, USA) with analytical column ReproSil-Pur (ODS-3, 100 × 4 mm, 3 µm) (Dr A. Maisch, GmbH, Ammerbuch, Germany) was used. The mobile phase for HPLC analysis was 0.1 M citrate-phosphate buffer containing 1.1 M octane sulfonic acid, 0.1 mM ethylenediaminetetraacetic acid (EDTA), and 9% solution of acetonitrile (pH = 3.0). Measurements were made using the electrochemical detector LC-4B (BAS, West Lafayette, IN, USA) on a glass–carbonic electrode (+0.85 V) against the reference electrode Ag/AgCl. The turnover of monoamines was expressed as the ratio of tissue concentrations of the primary acidic metabolite (DOPAC, HVA, or 5-HIAA) to the parent amine (DA, or 5-HT). Samples from all animals were processed in parallel on the same day for each brain structure. We used a solution that contained 3,4-dihydroxy benzylamine (DHBA) (Sigma, Ronkonkoma, NY, USA), NA (Bioanalytical Systems, Inc., West Lafayette, IN, USA), DA (Sigma, USA), DOPAC (Sigma, USA), HVA (Sigma, USA), 3-methoxytyramine (3-MT) (Sigma, USA), 5-HT (Calbiochem, San Diego, CA, USA), and 5-HIAA (Fluka, Radnor, PA, USA) as a standard at a concentration of 500 pmol/mL. The “internal standard” method was used to determine the levels of monoamines and their metabolites in brain structures as the ratio of the peak areas in the standard mixture and sample (nmol/g wet tissue). Samples were registered using the hardware-software complex Multichrom 1.5 (Ampersand, Moscow, Russia). All the reagents used for the analysis were of high purity.

### 4.4. Statistical Analysis

Data were analyzed using the program “STATISTICA Release 7”. A two-way or one-way analysis of variance (ANOVA) with the Newman–Keuls post hoc test, as well as a non-parametric equivalent, the Kruskal–Wallis H test (one-way ANOVA by ranks) with the multiple comparisons of mean ranks for all groups as a post hoc test, and the Mann–Whitney U test were used when appropriate. The factors ‘strain’ (Wistar, WAG/Rij) and ‘sex’ (males, females) were used as independent variables. The measures characterizing anxiety, behavioural depression-like symptoms, monoamines and their metabolites content in brain structures were treated as dependent variables. Because body weight is a variable that might affect sucrose consumption or preference, analysis of covariance (ANCOVA) was conducted, with strain and sex as independent variables and body weight of rats as a covariate. In addition to ANCOVA, Pearson correlation coefficients were computed between body weight and sucrose test measures. Effects were considered significant at *p* < 0.05. Homogeneity and normality of variance were assessed by Levene’s test, the Kolmogorov–Smirnov test, and Lilliefors’ test, respectively. GraphPad Prism 8 software was used to plot the figures.

## 5. Conclusions

Despite the high incidence of depression in human epilepsy and the well-established sex differences in depression presentation, sex differences in depression in preclinical epilepsy models, as well as their mechanisms, remain poorly understood.

In the present study, sex differences were found in the manifestation of depression comorbid with absence epilepsy in a preclinical WAG/Rij rat model. As far as we know, this study is the first to show differences in brain neurochemistry in male and female WAG/Rij rats with different presentations of depression-like pathology.

The results of behavioural studies indicate that female WAG/Rij, like male WAG/Rij, exhibit distinct symptoms of depression in the forced swimming, sucrose preference, and splash tests. However, female WAG/Rij, in contrast to male WAG/Rij, exhibit more pronounced signs of increased anxiety, anhedonia, and a predisposition to hyperactivity in a novel, stressful environment. These behavioural symptoms are characteristic of a subtype of depressive disorders, such as anxious depression.

In the neurochemical studies, significant differences were found in the content of DA and its metabolites in brain structures responsible for the manifestation of depression-like behavioural symptoms (the prefrontal cortex, nucleus accumbens, striatum, and hypothalamus). In WAG/Rij males compared to Wistar males, decreased levels of DA and its metabolites were observed in the prefrontal cortex, nucleus accumbens, striatum, and hypothalamus. This indicates a reduced DAergic tone in the brain. Compared to Wistar females, WAG/Rij females, like males, also showed reduced activity in the DAergic brain system. This means that similar alterations in the functional activity of the DAergic brain system in male and female WAG/Rij rats compared to control Wistar rats may underlie a similar depressive-like behavioural profile described in this study. However, in WAG/Rij females, compared to males, lower levels of DA and 3-MT, a marker of released DA, were found in the nucleus accumbens, a key brain structure known to be involved in reward, motivation, and depression-like behaviours. These sex-related biochemical changes may explain the more pronounced anhedonia (in sucrose consumption/preference and splash tests) in female WAG/Rij rats compared to WAG/Rij males. Sex differences not only in the DArgic but also in the 5-HTergic system of the brain were found in WAG/Rij rats. It is suggested that the dysfunction of not only the DAergic, but also the 5-HTergic brain system may underlie the anxious depression phenotype in female WAG/Rij rats. Whether sex differences in the content of monoamines and their metabolites in the brain structures of WAG/Rij rats are related to sex differences in the expression of monoaminergic genes await investigation.

A more comprehensive understanding of sex differences in the functioning of monoaminergic brain systems, primarily DAergic and 5-HTergic, is particularly important in the development of more effective, pathogenetically based therapeutics for the treatment of depressive disorders comorbid with epilepsy in individuals of both sexes.

## Figures and Tables

**Table 1:** Sex differences in monoamines and their metabolites content in brain structures of WAG/Rij and Wistar rats.

Biochemical Measures, nmol/g Tissue	Groups	Brain Structures				
		Prefrontal Cortex	Nucleus Accumbens	Striatum	Hypothalamus	Hippocampus
NA	WS-m WS-f WR-m WR-f	2.10 ± 0.08 1.91 ± 0.06 2.33 ± 0.11 # 2.05 ± 0.06 *	2.68 ± 0.26 2.76 ± 0.39 4.28 ± 0.42 # 3.69 ± 0.55	0.55 ± 0.05 0.65 ± 0.09 0.81 ± 0.08 0.55 ± 0.04 *	7.29 ± 0.17 6.39 ± 0.21 ** 8.27 ± 0.24 ## 7.09 ± 0.17 *#	2.63 ± 0.74 2.61 ± 0.10 2.86 ± 0.13 2.64 ± 0.11
DOPAC	WS-m WS-f WR-m WR-f	0.14 ± 0.02 0.18 ± 0.02 0.16 ± 0.02 0.17 ± 0.01	4.44 ± 0.26 3.69 ± 0.11 * 3.79 ± 0.20 # 3.31 ± 0.19	5.00 ± 0.22 4.48 ± 0.13 * 3.87 ± 0.13 ## 3.77 ± 0.15 ##	0.32 ± 0.02 0.28 ± 0.02 * 0.23 ± 0.01 # 0.23 ± 0.01 #	0.21 ± 0.02 0.19 ± 0.02 0.18 ± 0.01 0.17 ± 0.01
DA	WS-m WS-f WR-m WR-f	0.28 ± 0.03 0.27 ± 0.03 0.17 ± 0.02 # 0.22 ± 0.02	41.61 ± 1.51 35.46 ± 0.80 ** 34.84 ± 1.86 ## 30.44 ± 1.62 *#	75.94 ± 1.73 67.75 ± 2.21 ** 63.87 ± 1.91 ## 60.72 ± 1.98 #	2.52 ± 0.22 1.94 ± 0.13 ** 1.72 ± 0.07 ## 1.84 ± 0.09	0.09 ± 0.06 0.07 ± 0.02 0.04 ± 0.01 0.03 ± 0.01
HVA	WS-m WS-f WR-m WR-f	0.30 ± 0.05 0.26 ± 0.04 0.27 ± 0.07 0.21 ± 0.03	2.03 ± 0.14 1.66 ± 0.12 * 1.65 ± 0.12 # 1.40 ± 0.08	3.34 ± 0.16 3.04 ± 0.17 2.57 ± 0.07 ## 2.34 ± 0.12 ##	0.12 ± 0.01 0.10 ± 0.01 0.12 ± 0.04 0.11 ± 0.02	0.09 ± 0.01 0.10 ± 0.01 0.12 ± 0.01 0.11 ± 0.01
3-MT	WS-m WS-f WR-m WR-f	0.20 ± 0.04 0.20 ± 0.03 0.19 ± 0.04 0.20 ± 0.03	0.67 ± 0.07 0.47 ± 0.02 * 0.78 ± 0.06 0.57 ± 0.05 *	1.24 ± 0.06 0.96 ± 0.05 * 1.41 ± 0.09 1.18 ± 0.05 *#	0.04 ± 0.01 0.03 ± 0.01 0.04 ± 0.01 0.06 ± 0.01 #	0.05 ± 0.01 0.10 ± 0.04 0.07 ± 0.01 0.07 ± 0.01
DOPAC/DA	WS-m WS-f WR-m WR-f	0.66 ± 0.13 0.82 ± 0.11 1.29 ± 0.34 0.89 ± 0.10	0.12 ± 0.004 0.12 ± 0.003 0.10 ± 0.003 0.10 ± 0.003	0.07 ± 0.001 0.07 ± 0.001 0.06 ± 0.001 0.07 ± 0.001	0.14 ± 0.01 0.16 ± 0.01 0.15 ± 0.01 0.14 ± 0.01	11.00 ± 3.0 8.19 ± 2.77 8.56 ± 1.73 9.62 ±1.87
HVA/DA	WS-m WS-f WR-m WR-f	0.72 ± 0.20 0.57 ± 0.11 0.97 ± 0.30 0.52 ± 0.08	0.032 ± 0.001 0.031 ± 0.001 0.027 ± 0.001 0.030 ± 0.001	0.02 ± 0.001 0.02 ± 0.001 0.02 ± 0.001 0.02 ± 0.001	0.03 ± 0.01 0.03 ± 0.01 0.04 ± 0.02 0.03 ± 0.01	3.04 ± 1.04 2.49 ± 1.01 2.84 ± 0.62 5.14 ± 1.64
5-HT	WS-m WS-f WR-m WR-f	4.67 ± 0.18 4.55 ± 0.18 4.21 ± 0.24 4.27 ± 0.11	7.81 ± 0.24 8.32 ± 0.29 8.55 ± 0.24 8.35 ± 0.51	5.10 ± 0.11 5.34 ± 0.21 5.22 ± 0.10 5.18 ± 0.16	7.81 ± 0.24 8.32 ± 0.29 8.55 ± 0.24 8.35 ± 0.51	3.67 ± 0.14 3.93 ± 0.14 3.30 ± 0.10 # 3.21 ± 0.13 #
5-HIAA	WS-m WS-f WR-m WR-f	1.07 ± 0.03 0.98 ± 0.06 1.16 ± 0.05 1.17 ± 0.05 #	2.97 ± 0.13 3.09 ± 0.08 3.48 ± 0.14 # 3.36 ± 0.12	2.23 ± 0.09 3.44 ± 0.12 3.58 ± 0.11 3.40 ± 0.12	2.78 ± 0.14 2.83 ± 0.13 2.87 ± 0.20 2.86 ± 0.17	1.98 ± 0.08 2.30 ± 0.05 * 2.23 ± 0.07 # 2.22 ± 0.07
5-HIAA/5-HT	WS-m WS-f WR-m WR-f	0.28 ± 0.01 0.26 ± 0.01 0.34 ± 0.02 # 0.33 ± 0.01 #	0.47 ± 0.02 0.46 ± 0.01 0.50 ± 0.02 0.53 ± 0.02 #	0.81 ± 0.03 0.82 ± 0.03 0.87 ± 0.02 0.83 ± 0.01	0.47 ± 0.01 0.48 ± 0.01 0.50 ± 0.02 0.50 ± 0.01	0.65 ± 0.02 0.71 ± 0.02 0.82 ± 0.02 ## 0.84 ± 0.03 ##

Values are the mean ± standard error of the mean (M ± S.E.M.). NA—noradrenaline, DA—dopamine, DOPAC—3,4-dihydroxyphenylacetic acid, HVA—homovanillic acid, 3-MT—3-methoxytyramine, 5-HT—serotonin, 5-HIAA—5-hydroxy indole acetic acid. WS-m—Wistar males (*n* = 12), WS-f—Wistar females (*n* = 13), WR-m—WAG/Rij males (*n* = 12), WR-f—WAG/Rij females (*n* = 13). *—*p <* 0.05, **—*p <* 0.01, females compared to males of the same strain; #—*p* < 0.05, ##—*p* < 0.01, WAG/Rij rats vs. Wistar rats of the same sex. Significant differences are highlighted in yellow.

**Table 2:** Schematic representation of the same changes in the neurochemical systems of the brain in male and female WAG/Rij rats compared with Wistar rats.

Brain Structures	Groups of Rats	
	WAG/Rij Males	WAG/Rij Females
	Dopaminergic system	
Nucleus accumbens	DA ↓	DA ↓
Striatum	DA ↓	DA ↓
	HVA ↓	HVA ↓
	DOPAC ↓	DOPAC ↓
Hypothalamus	DOPAC ↓	DOPAC ↓
	Serotonergic system	
Prefrontal cortex	5-HIAA/5-HT ↑	5-HIAA/5-HT ↑
Hippocampus	5-HT ↓	5-HT ↓
	5-HIAA/5-HT ↑	5-HIAA/5-HT ↑
	Noradrenergic system	
Hypothalamus	NA ↑	NA ↑

Downwards arrows—decreased, upwards arrows—increased.

## Data Availability

The experimental data/Supplementary Materials are fully presented in the manuscript. Additional information may be provided upon reasonable request.

## Supplementary Materials

The following supporting information can be downloaded at https://www.mdpi.com/article/10.3390/ijms27052154/s1.
