# Supplementary material for: The WAG/Rij Rat Model of Depression Comorbid with Absence Epilepsy: Sex Differences and Neurochemical Mechanisms
Source: Int J Mol Sci. 2026 Feb 25;27(5):2154. doi: 10.3390/ijms27052154 (PMC12984789; doi:10.3390/ijms27052154)
Supplement: Supplementary file 1 [file ijms-27-02154-s001.zip › ijms-4066048-supplementary.pdf]

Table S1. Post-hoc test results (*p*-values) for strain and sex differences in monoamines and their metabolite content in brain structures of Wistar and WAG/Rij rats

Brain structures	Biochemical measures	Groups being compared			
		WR-m /WS-m	WR-f/WS-f	WR-f/WR-m	WS-f/WS-m
PFC	NA	*p*(F)=0.049	NS	*p*(F)=0.049	NS
	DA	*p*(F)=0.021	NS	NS	NS
	5-HIAA	NS	*p*(F)=0.044	NS	NS
	5-HIAA/5-HT	*p*(F)=0.018	*p*(F)=0.008	NS	NS
NAc	NA	p(F)=0.049	NS	NS	NS
	DOPAC	p(F)=0.022	NS	NS	p(F)=0.025
	DA	p(F)=0.007	p(U)=0.017	p(F)=0.042	p(F)=0.006
	HVA	p(U)=0.049	NS	NS	p(F)=0.026
	3-MT	NS	NS	p(F)=0.010	p(F)=0.024
	5-HIAA	p(F)=0.021	NS	NS	NS
	5-HIAA/5-HT	NS	p(F)=0.033	NS	NS
STR	NA	NS	NS	p(F)=0.035	NS
	DOPAC	p(F)=0.001	p(F)=0.008	NS	p(F)=0.028
	DA	p(F)=0.0004	p(F)=0.041	NS	p(F)=0.005
	HVA	p(F)=0.001	p(F)=0.002	NS	NS
	3-MT	NS	p(F)=0.024	p(F)=0.034	p(F)=0.012
HYPO	NA	p(F)=0.001	p(F)=0.018	p(F)=0.0004	p(F)=0.008
	DOPAC	p(F)=0.0003	p(F)=0.024	NS	p(F)=0.038
	DA	p(F)=0.0009	NS	NS	p(F)=0.0039
	3-MT	NS	p(F)=0.018	NS	NS
HIPPO	5-HT	p(F)=0.049	p(F)=0.002	NS	NS
	5-HIAA	p(F)=0.034	NS	NS	p(F)=0.019
	5-HIAA/5-HT	p(F)=0.0001	p(F)=0.008	NS	NS

PFC – prefrontal cortex, NAc – nucleus accumbens, STR – striatum, HYPO – hypothalamus, HIPPO – hippocampus. *p*(F) – post-hoc Newman-Keuls test, *p*(U) – post-hoc Mann-Whitney U test, NS – non-significant differences.
